# Nurse-Led, Telephone-Based, Secondary Preventive Follow-Up after Stroke or Transient Ischemic Attack Improves Blood Pressure and LDL Cholesterol: Results from the First 12 Months of the Randomized, Controlled NAILED Stroke Risk Factor Trial

**DOI:** 10.1371/journal.pone.0139997

**Published:** 2015-10-16

**Authors:** Anna-Lotta Irewall, Joachim Ögren, Lisa Bergström, Katarina Laurell, Lars Söderström, Thomas Mooe

**Affiliations:** 1 Department of Public Health and Clinical Medicine, Östersund, Umeå University, Umeå, Sweden; 2 Department of Pharmacology and Clinical Neuroscience, Östersund, Umeå University, Umeå, Sweden; 3 Unit of Research, Development and Education, Region Jämtland Härjedalen, Östersund Hospital, Östersund, Sweden; University of Bologna, ITALY

## Abstract

**Background:**

Enhanced secondary preventive follow-up after stroke or transient ischemic attack (TIA) is necessary for improved adherence to recommendations regarding blood pressure (BP) and low-density lipoprotein cholesterol (LDL-C) levels. We investigated whether nurse-led, telephone-based follow-up was more efficient than usual care at improving BP and LDL-C levels at 12 months after hospital discharge.

**Methods:**

We randomized 537 patients to either nurse-led, telephone-based follow-up (intervention) or usual care (control). BP and LDL-C measurements were performed at 1 month (baseline) and 12 months post-discharge. Intervention group patients who did not meet target values at baseline received additional follow-up, including titration of medication and lifestyle counselling, to reach treatment goals (BP < 140/90 mmHg, LDL-C < 2.5 mmol/L).

**Results:**

At 12 months, mean systolic BP, diastolic BP and LDL-C was 3.3 (95% CI 0.3 to 6.3) mmHg, 2.3 mmHg (95% CI 0.5 to 4.2) and 0.3 mmol/L (95% CI 0.1 to 0.4) lower in the intervention group compared to controls. Among participants with values above the treatment goal at baseline, the difference in systolic BP and LDL-C was more pronounced (8.0 mmHg, 95% CI 4.0 to 12.1, and 0.6 mmol/L, 95% CI 0.4 to 0.9). A larger proportion of the intervention group reached the treatment goal for systolic BP (68.5 vs. 56.8%, *p* = 0.008) and LDL-C (69.7% vs. 50.4%, *p* < 0.001).

**Conclusions:**

Nurse-led, telephone-based secondary preventive follow-up, including medication adjustment, was significantly more efficient than usual care at improving BP and LDL-C levels by 12 months post-discharge.

**Trial Registration:**

ISRCTN Registry ISRCTN23868518

## Introduction

Stroke is a major cause of morbidity and mortality worldwide [1] and it is well-established that stroke survivors are at high risk of suffering subsequent vascular events [2–4]. From a global perspective, the prevalence of stroke survivors has increased over the last two decades [1], increasing the need for secondary prevention.

Antihypertensive and lipid-lowering treatment after stroke to prevent vascular events is supported by the results of randomized controlled trials [5, 6] and is recommended by clinical guidelines [7, 8]. However, observational follow-up data from different countries show that the proportion of patients who achieve the recommended target values for blood pressure (BP) and low-density lipoprotein cholesterol (LDL-C) may be as low as 24–38% [9–11] and 14–24% [9, 10], respectively.

Various strategies to improve control of modifiable risk factors after stroke or TIA have been evaluated in randomized controlled trials; however, very few to date have resulted in any significant improvement compared to usual care [12]. A systematic review of interventions intended to improve BP values in patients with essential hypertension concluded that 1) improved BP control generally requires a systematic approach, including both regular BP monitoring and review of pharmacological treatment, and that 2) secondary preventive follow-up programs that are led by nurses or pharmacists show promising but heterogeneous results [13]. In studies in which participant populations are restricted to stroke and TIA patients, follow-up programs that are mainly led by nurses, as compared to the usual follow-up care have, so far, achieved modest results, mostly without statistical significance, in terms of improved BP and LDL-C levels [12, 14–20]. Most of these studies have, however, been rather small [12, 14–16, 18, 19] and have not included pharmacological titration as part of the intervention [14, 15, 18–20].

The NAILED stroke risk factor trial is an on-going population-based, randomized controlled trial to test the hypothesis that nurse-led, telephone-based, secondary preventive follow-up, including pharmacological titration, may be a more effective approach than usual care with respect to improving modifiable risk factors after stroke or TIA [21]. The objective of our present study was to investigate whether the NAILED trial intervention would prove to be more efficient than usual care at improving BP values and LDL-C levels at 12 months after hospital discharge.

## Materials and Methods

### Study design

The NAILED stroke risk factor trial was designed to be an open, population-based, randomized controlled trial comprising two parallel groups with an allocation ratio of 1:1. The design of the trial and the intervention evaluated is described in further detail in the published study protocol [21] and we will describe it below in brief.

### Outcomes

The primary outcome of our present study was to determine the mean difference in seated systolic blood pressure (SBP) between patients in the two groups at 12 months post-discharge. We analysed the following variables as secondary outcomes: the mean differences in diastolic blood pressure (DBP) and LDL-C between groups, differences in the proportion of patients who reached the target values for each measure, and changes in SBP, DBP, and LDL-C between baseline and 12 months within each group. Measurement of outcome variables at 12 months was pre-specified in the study protocol, but since the primary outcome of the NAILED stroke risk factor trial is the seated systolic blood pressure measured after 36 months of follow-up, the analyses of our present report are exploratory.

### Sample size

To reliably detect a difference between groups in mean SBP of 5 mmHg, study groups of 180 participants (standard deviation 19 mmHg, mean SBP 140 versus 135 mmHg, alpha 0.05 two-tailed, power 80%) were needed. To allow for drops-outs, we planned for study groups of at least 200 participants.

### Setting and eligibility criteria

We recruited participants at Östersund Hospital between Jan 1^st^, 2010 and June 30^th^, 2012. The hospital is the only hospital in a geographically large, rural area in central Sweden that is inhabited by approximately 125,000 people.

In order to identify all of the patients who were admitted to the hospital and subsequently diagnosed with an acute stroke or TIA, we conducted daily review of the hospital records of all patients who had computed tomography (CT) brain scans. In addition, we checked the stroke unit on a daily basis to catch any patient who was diagnosed with stroke or TIA without undergoing a CT scan.

We considered all of the identified stroke and TIA patients who were admitted to the hospital during the inclusion period to be eligible. However, participants in other, concurrent trials and patients who were considered unable to participate in the study follow-up due to aphasia, cognitive impairment, impaired hearing, or severe, often terminal, disease had to be excluded.

### Randomization and blinding

Eligible patients who consented to participate and who did not meet any of the exclusion criteria were randomly assigned to the intervention or the control group. The randomized allocation sequence was computer generated in blocks of four and was stratified for sex and for degree of disability (modified Rankin Scale <3 or ≥3). The resulting group allocation was not blinded to participants, the study team or other caregivers.

### Data collection

We collected baseline data, including prevalence of cardiovascular risk factors, prior cardiovascular events, and comorbidity, by patient interviews in-hospital and data were further supplemented by review of the medical records.

Baseline and follow-up measurements of BP and blood lipids at 1 and 12 months post-discharge, respectively, was performed by a health care professional at the patients’ closest health care facility and reported to the study team. Shortly after the baseline and 12-month BP and blood lipid measurements, a study nurse telephoned participants in both study groups and interviewed them about their sense of well-being, use of tobacco, level of physical activity, and compliance with recommended medical treatments.

In addition, we conducted a systematic review of patient medical records to retrospectively collect descriptive data on secondary preventive follow-up provided via primary care visits and at the hospital out-patient clinic during the first year after discharge.

### Intervention follow-up

For the intervention group, follow-up included telephone-based lifestyle counselling and assessment of pharmacological treatment. If the target value for BP and/or lipids was not met at the baseline measurement, the study nurse consulted a study physician for assessment and adjustment of the patient’s pharmacological treatment. Within approximately 4 weeks after a pharmacological adjustment, participants where called for a new measurement and the process was repeated if necessary. All pharmacological adjustments were individualized to the needs of the patient, not restricted to any fixed algorithm or protocol. Prescription of lipid-lowering therapy was restricted to patients who had ischemic events [7, 8].

### Secondary preventive follow-up in the control group

The control group participants received secondary preventive care according to local standard procedures, hence referred to as “usual care.” Telephone contact with participants in the control group did not include any lifestyle counselling or any pharmacological assessment. BP and LDL-C values from the study measurements were forwarded to the patient’s GP for assessment and no further action was taken by the study team.

Secondary preventive treatment was generally initiated in-hospital. Thereafter, the patients’ general practitioners (GPs) held primary responsibility for their care.

### Target values

For the purpose of this study, we considered a systolic blood pressure (SBP) <140 mmHg, a diastolic blood pressure (DBP) <90 mmHg, and an LDL-C value <2.5 mmol/L to be within target with respect to compliance with national guidelines.

### Definition of disease

The qualifying events, prior vascular events, and comorbid conditions refer to diagnoses made by clinical physicians. Stroke included both ischemic and hemorrhagic events, with the exception of subarachnoid hemorrhage. We defined prior ischemic heart disease to include previous acute myocardial infarction, percutaneous coronary intervention, and/or coronary artery bypass grafting.

### Statistical method

We performed the analysis in accordance with the intention-to-treat principle. Comparisons of baseline characteristics between groups (intervention vs. control, participants vs. lost to follow-up) were performed using independent sample *t*-tests and chi-square tests as appropriate. We used paired sample *t*-tests to evaluate changes in mean BP and LDL-C values between baseline and 12 months within a single group.

We calculated the adjusted mean differences between groups (intervention vs. control) in BP and LDL-C levels at 12 months using a general linear model adjusted for sex and degree of disability in order to reflect the stratified randomization [21].

A second general linear model was constructed to test the hypothesis that any difference detected between groups at 12 months might be primarily attributable to benefits affecting intervention group participants who had not reached the target at baseline. In addition to accounting for sex and degree of disability, this second model included 1) a binary indicator variable denoting participants as either above or below the target value at the baseline measurement and 2) an interaction variable between the same variable and the treatment group allocation.

Hemorrhagic stroke patients were not included in analyses concerning LDL-C, with the exception of the baseline table (Table 1).

**Table 1:** Baseline characteristics of participants who completed the 12 months follow-up.

	Intervention	Control
N (%)	241 (49.8)	243 (50.2)
Age, years ± SD	71.5 ± 11.1	70.1 ± 10.4
Male, N (%)	137 (56.8)	139 (57.2)
TIA, N (%)	89 (36.9)	89 (36.6)
Ischemic stroke, N (%)	143 (59.3)	146 (60.1)
Hemorrhagic stroke, N (%)	9 (3.7)	8 (3.3)
mRS <3, N (%)	203 (83.8)	216 (88.9)
Diabetes mellitus, N (%)	40 (16.6)	46 (18.9)
Atrial fibrillation, N (%)	39 (16.2)	39 (16.0)
Congestive heart failure, N (%)	10 (4.1)	7 (2.9)
Previous stroke, N (%)	41 (17.0)	32 (13.2)
Previous IHD, N (%)	30 (12.4)	29 (11.9)
Current smoker, N (%)	28 (11.6)	41 (16.9)
1 month after discharge (baseline):		
Antihypertensive drug (≥1), N (%)	177 (73.4)	186 (76.5)
Lipid-lowering drug, N (%)	191 (79.3)	193 (79.4)
Anti-platelet drug, N (%)	191 (79.3)	199 (81.9)
OAC, N (%)	36 (14.9)	31 (12.8)

The intervention and control group did not differ significantly in any of the baseline characteristics. mRS, modified Rankin Scale; IHD, ischemic heart disease; OAC, oral anticoagulation; SBP, systolic blood pressure; DBP, diastolic blood pressure; LDL-C, low-density lipoprotein cholesterol; SD, standard deviation.

All analyses were performed using SPSS software, version 22.0, and we defined the significance threshold at the level of p = 0.05.

### Ethics

The study was approved by the Regional Ethics Committee of Umeå University, Umeå, on Oct 28, 2009. All participants signed an informed, written consent document.

### Trial registration

The NAILED stroke risk factor trial is registered in the ISRCTN registry (ISRCTN23868518). The ICMJE strict requirement of prospective registration of clinical trails came to our attention when the recruitment had already begun. The study was therefore retrospectively registered. The authors confirm that all on-going and related trials for this intervention are now registered.

### Updates to the original study protocol

Before patient enrolment, a few minor adjustments to the protocol was made compared to the protocol approved by the ethics committee (S1 Protocol): 1) Patient enrolment begun on Jan 1^st^ 2010 instead of Jan 4^th^, 2010. 2) We changed the time range from hospital discharge to the first follow-up from 6–10 weeks to 1 month. 3) We reprioritized the outcome variables of the study so that the absolute difference in risk factor values became the primary outcome. 4) We used stratified randomization in order to assure equal distribution of clinically important covariates. 5) We revised the statistical method for outcome variables in order to reflect the stratified randomization. In addition, minor updates to the risk factor target values were made in order to follow new guidelines published during the course of study. None of these adjustments required reapproval from the ethics committee.

## Results

**Fig 1:**
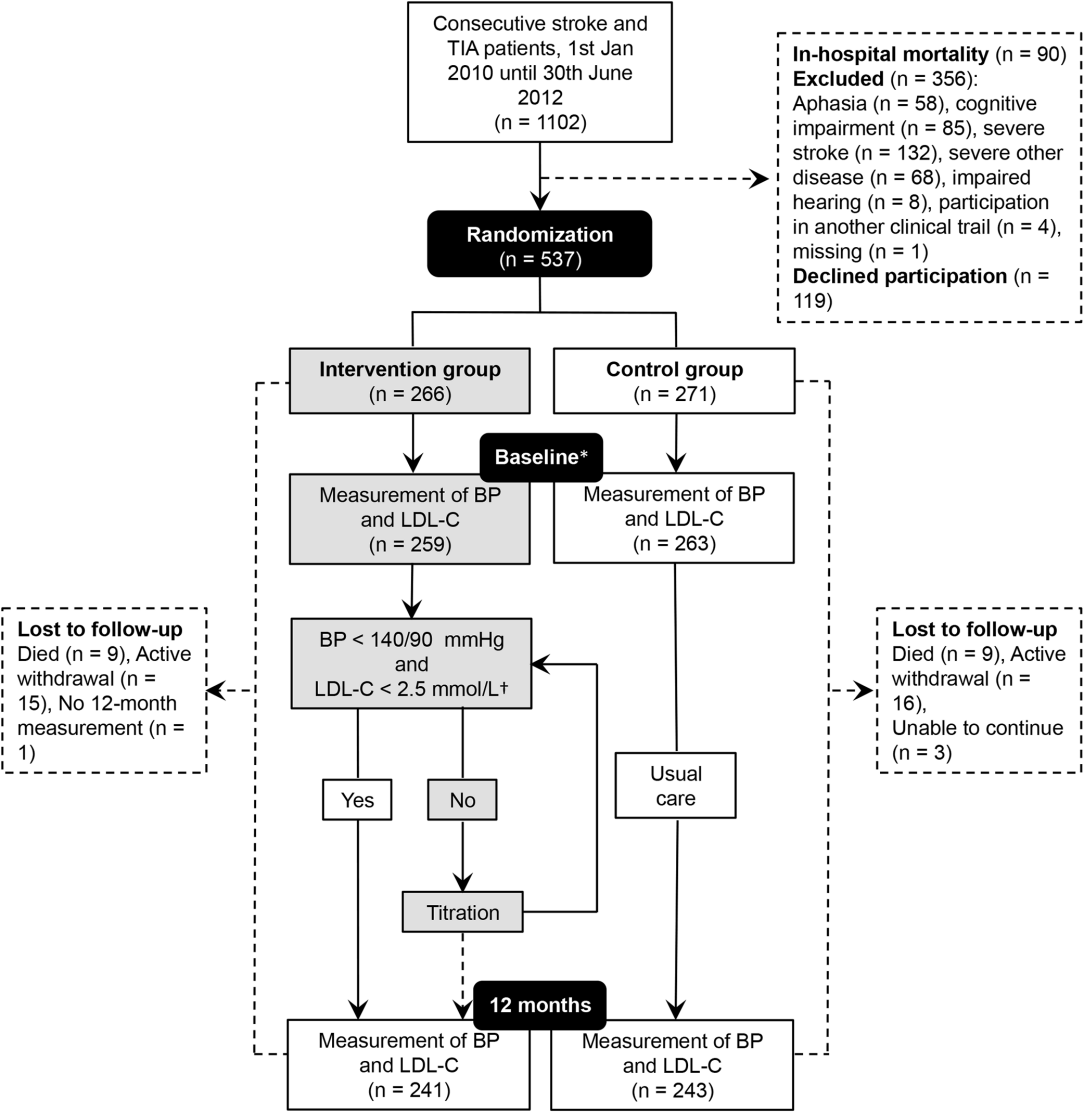
Study design flow-chart. Participants who were lost to follow-up during the 12-month period were not included in the final analysis due to missing values for BP and LDL-C from the 12-month follow-up measurement. BP, blood pressure; LDL-C, low-density lipoprotein cholesterol; TIA, Transient Ischemic Attack. * 1 month post-discharge. † The LDL-C target value only concerned participants who had experienced an ischemic stroke.

The flow of participants is illustrated in Fig 1. Out of the 537 patients originally randomized, a total of 484 participants, 276 male (57.0%) and 208 female (43.0%), completed the 12-month follow-up and were included in our final analysis. Stroke (63.2%, n = 306) was more common compared to TIA (36.8%, n = 178) and the mean patient age overall was 70.8 (±10.7). Detailed baseline characteristics are given in Table 1. The participants who were lost to follow-up (n = 53) were significantly older, more often female, and compared to the participants who completed the 12-month follow-up they showed a poorer functional status upon hospital discharge, with a higher prevalence of modified Rankin Scale ≥ 3, congestive heart failure and atrial fibrillation.

### Secondary preventive follow-up

In Table 2 we provide a summary of the health-care contacts made with participants in the intervention and control groups from baseline (1 month after discharge) until the 12-month follow-up.

**Table 2:** Secondary preventive follow-up from baseline measurement (1 month after discharge) until 12 months after discharge.

	Intervention			Control		
		BP <140/90 mmHg and LDL-C <2.5 mmol/L at baseline?			BP <140/90 mmHg and LDL-C <2.5 mmol/L at baseline?	
	All	Yes	No	All	Yes	No
Return visit to the hospital out-patient clinic, yes % (n)	16.2 (39)			14.0 (34)		
In contact with a primary care center, yes* % (n)	97.1 (234)			95.1 (231)		
No of contacts with a physician, median (IQR)	3.0 (1.0–6.0)	4.0 (2.0–6.0)	3.0 (1.0–5.0)	3.5 (1.0–6.0)	3.0 (1.5–7.0)	4.0 (1.0–6.0)
No of BP and/or LDL-C evaluations†, median (IQR)	1.0 (0.0–2.0)	1.0 (0.0–2.0)	1.0 (0.0–2.0)	3.0 (1.0–4.0)	2.0 (1.0–3.0)	3.0 (2.0–4.0)
The NAILED study follow-up:						
No of contacts with a study nurse, median (IQR)	4.0 (3.0–7.0)	2.0 (2.0–2.3)	6.0 (4.0–8.0)	2.0 (2.0–2.0)	2.0 (2.0–2.0)	2.0 (2.0–2.0)
No of BP and/or LDL-C evaluations†, median (IQR)	2.0 (1.0–3.0)	1.0 (1.0–1.0)	3.0 (2.0–4.0)	-	-	-

IQR, interquartile range; BP, blood pressure; LDL-C, low-density lipoprotein cholesterol.

*Indicates at least one contact with a health professional at a primary care center in addition to the measurements performed for the study.

†All BP or LDL-C measurements documented in the medical record (BP measurements performed in situations of acute illness are not included).

### BP at 12-month follow-up

At the 12-month follow-up, both the mean SBP (-5.7 mmHg, 95% CI -8.3 to -3.0, p) and the mean DBP (-2.2 mmHg, 95% CI -3.8 to -0.7) had decreased significantly among patients in the intervention group, whereas no significant change in either mean SBP or DBP was observed among controls (Table 3). The mean difference in SBP and DBP between the two groups at the 12-month follow-up, adjusted for sex and for degree of disability, was 3.3 (95% CI 0.3 to 6.3) mmHg and 2.3 (95% CI 0.5 to 4.2) mmHg.

**Table 3:** SBP, DBP and LDL-C values at baseline, end of titration and at 12 months after discharge.

	Intervention			Control		
	Baseline	End of titration*	12 months	Baseline	12 months	Adjusted difference (95% CI) between groups at 12 months^†^
SBP (mmHg), mean (± SD)	137.5 (17.1)	126.9 (9.5)^‡^	131.9 (15.7)	136.9 (19.2)^§^	135.0 (17.5)	3.3 (0.3–6.3)
SBP <140 mmHg, % (n)	54.4 (131)	92.5 (222)	68.5 (165)	52.7 (127)	56.8 (138)	
Unadjusted mean (95% CI) change from last measurement		10.5 (8.6–12.4)	5.0 (3.0–6.9)		1.9 (-0.6–4.4)	
DBP (mmHg), mean (± SD)	79.5 (10.9)	73.9 (8.9)^‡^	77.3 (10.3)	79.3^§^ (10.5)	79.6 (10.5)	2.3 (0.5–4.2)
DBP <90 mmHg, % (n)	79.7 (192)	98.8 (237)	84.6 (204)	80.5 (194)	81.1 (197)	
Unadjusted mean (95% CI) change from last measurement		5.6 (4.3–6.9)	3.5 (2.2–4.8)		0.3 (-1.2–1.7)	
LDL-C (mmol/L), mean (± SD)**	2.5 (0.8)	2.0 (0.4)	2.3 (0.7)^‡^	2.5 (0.8)^§^	2.6 (0.9)^††^	0.3 (0.1–0.4)
LDL-C <2,5 mmol/L, % (n)	55.6 (129)	94.8 (220)	69.7 (161)	57.1 (133)	50.4 (115)	
Unadjusted mean (95% CI) change from last measurement		0.5 (0.4–0.6)	0.3 (0.3–0.4)		0.1 (0.0–0.2)	

There were no significant differences between the intervention group and the control group at baseline. SBP, systolic blood pressure; DBP, diastolic blood pressure; LDL-C, low-density lipoprotein cholesterol.

* The SBP/DBP/LDL-C values measured at the conclusion of the intensified follow-up in the intervention group. Blood pressure and LDL-C measurements for intervention group participants with values below target at baseline were not repeated until the 12 months follow-up. Indicated values were therefor calculated assuming that blood pressure and LDL-C values that were below target at baseline remained unchanged.

^†^ Adjusted for sex and for degree of disability.

^‡^ Missing value for 1 intervention group participant.

^§^ Missing value for 2 control group participants.

** The LDL-C analyses does not include participants with a hemorrhagic stroke as the qualifying event.

^††^ Missing value for 7 control group participants

**Fig 2:**
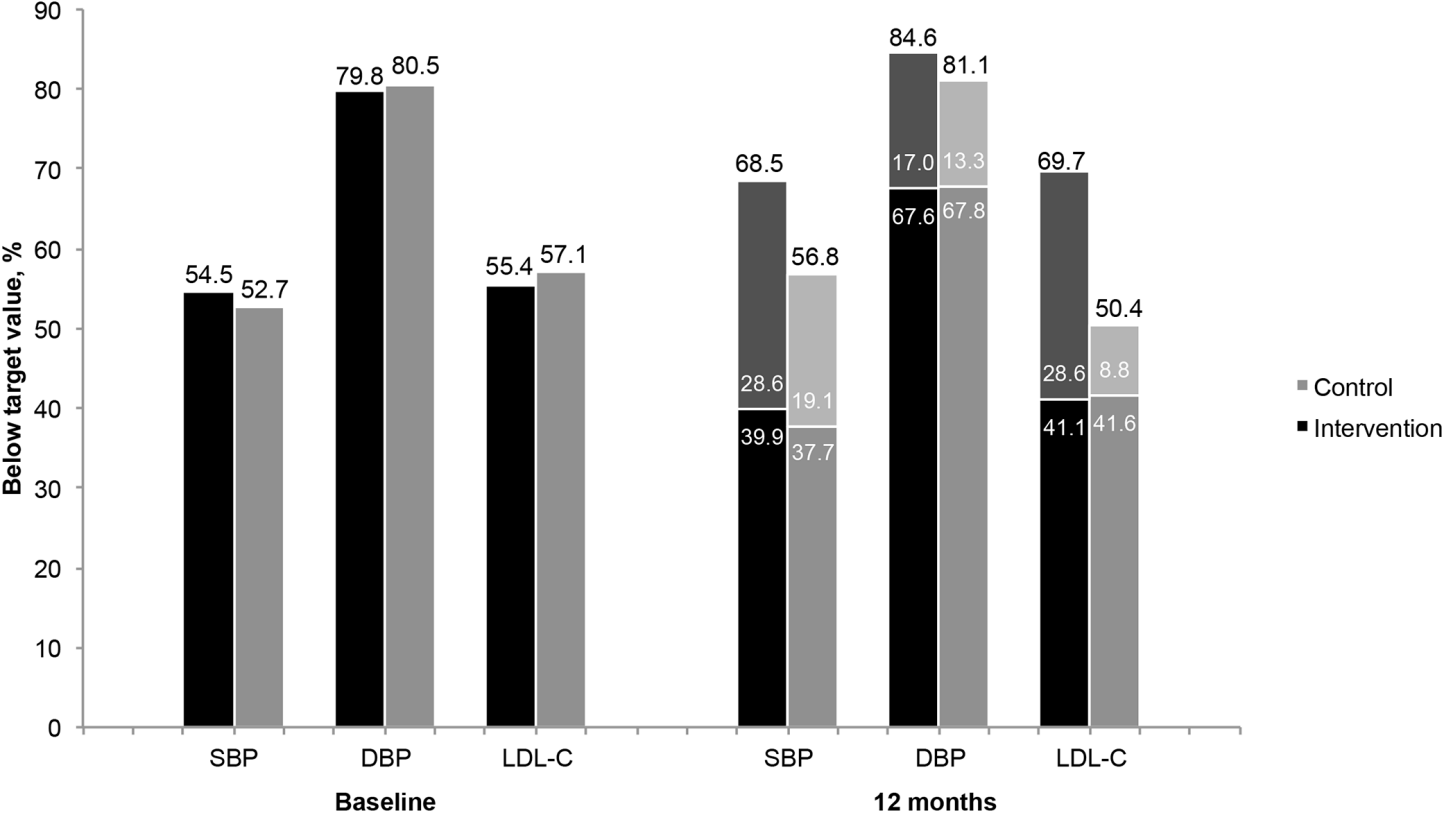
Participants with SBP, DBP and LDL-C below the target level at baseline and at 12 months. The lightly shaded portion of each 12 months stack represents the proportion of patients whose values changed from above target at baseline to below target at 12 months, whereas the dark shaded portion represents the proportion whose values remained below the target from baseline to 12 months. The differences observed between the intervention group and control group in the total proportion of participants with values below target at 12 months were significant with respect to SBP (*p* = 0.008) and LDL-C (*p* < 0.001). SBP, systolic blood pressure; DBP, diastolic blood pressure; LDL-C, low-density lipoprotein cholesterol.

Compared to the control group, a significantly larger proportion of participants in the intervention group reached the SBP target value (absolute difference of 11.7%, 95% CI 3.1 to 20.1) at the 12-month follow-up (Fig 2).

### LDL-C levels at 12-month follow-up

At the 12-month follow-up, the mean LDL-C value in the intervention group had decreased by 0.2 mmol/L (95% CI 0.1 to 0.3), whereas a significant increase (0.1 mmol/L, 95% CI 0.0 to 0.2) was observed among controls. The mean difference between the two groups at 12 months, adjusted for sex and for degree of disability, was 0.3 (95% CI 0.1 to 0.4) mmol/L. The target value was reached by 69.7% of the participants in the intervention group, compared to 50.4% of the control group (absolute difference 19.3%, 95% CI 10.3 to 27.8) (Fig 2).

### Interaction between treatment group allocation and baseline level of BP and LDL-C

**Fig 3:**
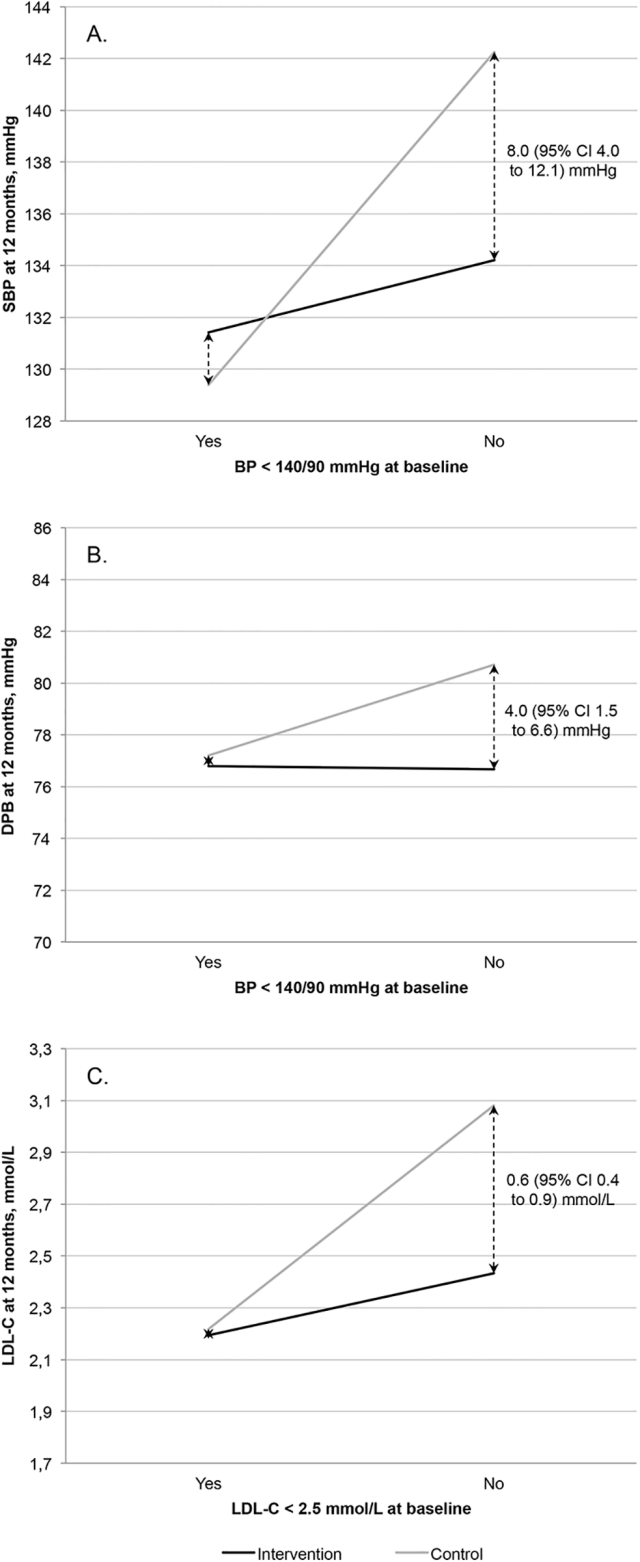
Effect of group allocation and baseline levels on the 12-month adjusted mean SBP, DBP and LDL-C. Effect of the interaction between group allocation and the baseline level of BP or LDL-C on the 12-month adjusted mean (A) SBP (*p* = 0.001), (B) DBP (*p* = 0.054), or (C) LDL-C (*p* < 0.001) value. BP, blood pressure; SBP, systolic blood pressure; DBP, diastolic blood pressure; LDL-C, low-density lipoprotein cholesterol.

Group allocation (intervention vs. control) and baseline values for BP/LDL-C (participants found above or below the target value) showed a significant interaction effect with the SBP (*p* = 0.001) and LDL-C (*p* < 0.001) levels measured at the 12-month follow-up, respectively (Fig 3). In the subgroup of participants who had a BP measurement above the target value at baseline, the adjusted mean SBP in the intervention group at 12 months was 8.0 (95% CI 4.0 to 12.1) mmHg lower compared to the control group. In the corresponding analysis for LDL-C, the adjusted mean at 12 months was 0.6 (95% CI 0.4 to 0.9) mmol/L lower. Conversely, no significant differences in SBP or LDL-C levels were observed at the 12-month follow-up between participants in the intervention group and control group whose values were below the respective targets at baseline.

### Maintenance of SBP and LDL-C values over time

Among the intervention group participants whose SBP and LDL-C were above the target values at baseline, treatment targets were achieved by 84.4% (92/109) and 90.3% (93/103) at the end of the intensified follow-up period. At the 12-month follow-up measurement, however, these proportions had significantly decreased to 62.7% (69/110) and 64.1% (66/103), respectively (absolute differences: SBP 21.7%, 95% CI 10.1 to 32.6; LDL-C 26.2%, 95% CI 15.0 to 36.8). A similar pattern was observed among the intervention and control group participants whose values were below the targets at baseline (Fig 2).

## Discussion

In this study, we found that nurse-led, telephone-based, secondary preventive follow-up improved BP and LDL-C levels and increased the proportion of patients who achieved the target values at 12 months post-discharge. Among participants whose values were above the targets at baseline, the intervention strategy resulted in mean SBP and LDL-C levels that were 8.0 mmHg and 0.6 mmol/L lower than the levels resulting from usual care. The overall differences between the intervention and control groups were diluted due to the inclusion of participants with risk factor levels that were already within the target range at baseline. This study design allowed us to demonstrate two additional findings of potential importance with respect to implementation of secondary preventive care: 1) About one-fourth of participants who had risk factor values within the target range at baseline were found above the target level at 12 months and 2) the proportion of intervention group participants achieving target values after intensified follow-up and pharmacological titration had decreased by about one-fourth at 12 months. This indicates that active monitoring of risk factors needs to extend beyond 12 months to maintain adequate risk factor control.

Improving the control of modifiable risk factors is necessary to reduce the incidence of recurrent vascular events and the resulting mortality. As health care resources, including access to stroke treatment and secondary preventive drugs, vary widely across different parts of the world, different regions face varying challenges in the improvement of secondary preventive care [22]. According to data from the Swedish stroke register, Riksstroke, Sweden has made progress in initiation of secondary preventive treatment [23], but the extent to which set treatment targets are reached and maintained on a population level is unknown. In our study population, the proportions of patients treated with different secondary preventive drugs at baseline were similar to the proportions in Riksstroke [24]. Despite this high adherence to recommended therapy, only about half of the population had achieved the recommended BP and LDL-C target values 1 month after hospital discharge. In the control group, these proportions did not improve over the 12 months following, even though most patients within that group had been in contact with their GP on average 3.5 times, and had their BP and/or LDL-C measured and evaluated on an average of 3 occasions during that year. Similar findings were described in a Danish cohort study. In this stroke and TIA population only 38% had a BP<140/90 mmHg at 12 months post-discharge. At 12 months, the proportion of patients on antihypertensive treatment had not increased compared to baseline and even among the 50% of the population who had their BP evaluated on at least 3 occasions during the 12 month period, only 34% had a BP <140/90 mmHg [25]. This emphasizes the need for improved secondary preventive follow-up to reach the full potential of available pharmacological treatment.

In our study, the total frequency of BP and LDL-C evaluations performed in the intervention group was very similar to the control group, suggesting that the number of follow-ups is not the single determinant of a successful program. A structured, out-reaching framework with capacity of systematic extra follow-up among those not reaching targets are, however, probably essential components according to our results.

Since human as well as monetary resources are limited within healthcare, increased involvement of various healthcare professionals other than physicians might be a cost effective way of improving secondary preventive care. Our study shows that secondary preventive follow-up delivered by nurses can be more efficient than usual care in terms of risk factor control. Our results do, however, not propose superiority of nurses over other healthcare professionals in this context and the question of cost effectiveness will be a matter of future analyses.

With few exceptions [19], previous studies of mainly nurse-led follow-up in stroke and TIA patients have failed to significantly improve BP compared to usual care [12, 14–18, 20]. In contrast to most previous studies [14, 15, 18–20], the NAILED intervention included pharmacological titration as part of the intensive follow-up given in response to elevated BP/LDL-C levels. Instead of direct adjustment of medication, a common strategy in previous studies was to refer participants to a physician not involved in the study team, either by sending information directly to the physician [14] or by advising the participant to contact the physician single-handed [15, 18–20]. One study found that even though the advice to see a GP was commonly followed by the participant, only one third of the patients had their hypertensive medication adjusted [15]. Similarly, a recently published observational study conducted among American veterans, showed that less than two thirds of all BP evaluations where intensified treatment was indicated led to altered pharmacological treatment [26]. Therapeutic inertia, defined as the physician’s decision not to intensify pharmacological treatment despite measurement of patient risk factors above recommended levels, has been previously recognized as an important barrier to improving adherence to clinical guidelines [27, 28]. Systematic pharmacological titration might, therefore, be a key component of a more effective secondary preventive follow-up program. This is supported by the recently published PREVENTION study, in which follow-up conducted by a pharmacist who was authorized to prescribe drugs was found to be significantly more effective than follow-up performed by nurses who lacked this authority [29].

### Strengths and weaknesses

The intervention approach tested in the NAILED stroke risk factor trial was designed to be broadly implementable in clinical practice. To best mimic a real clinical setting, each patient who fulfilled the basic inclusion criteria was assessed for enrolment based only on their ability to take part in the follow-up program. It could be argued that the absence of more precisely defined exclusion criteria might reduce the reproducibility of the study. However, on the contrary, we believe that the more pragmatic form of enrolment adopted in this study generated a study sample that is more representative of the general stroke and TIA patient population that could realistically be reached by this type of intervention. In addition, only a fairly small proportion of the eligible patients declined participation initially, and few ended their participation during this first year of follow-up. This increases the external validity of the results and should be considered a strength of the study.

We cannot rule out that the study setting, to some degree, influenced the secondary preventive treatment that was given to the control group. The study team provided the GPs with BP and LDL-C values, which might not have been measured otherwise. It is also possible that patients, by participating in the study, developed a heightened awareness and thereby became more prone to contact their GP for secondary preventive assessment. It is therefore reasonable to believe that any influence on the control group would have led to an underestimation, rather than an exaggeration, of the effect of the intervention.

## Conclusion

Evaluation of data from the first 12 months of the NAILED stroke risk factor trial showed that nurse-led, telephone-based follow-up, including titration of pharmacological treatment, was significantly more effective than the usual care provided to patients in improving control of both BP and LDL-C levels. Active intervention and monitoring of risk factors probably needs to extend beyond 12 months to maintain control over risk factors.

## Supporting Information

S1 CONSORT Checklist CONSORT checklist. (DOC)

S1 Protocol Original study protocol. Original study protocol as approved by the ethics committee.
(DOC)
